# Simultaneous Hand–Eye and Intrinsic Calibration of a Laser Profilometer Mounted on a Robot Arm

**DOI:** 10.3390/s21041037

**Published:** 2021-02-03

**Authors:** Urban Pavlovčič, Peter Arko, Matija Jezeršek

**Affiliations:** 1 Laboratory for Laser Techniques, Faculty of Mechanical Engineering, University of Ljubljana, Aškerčeva cesta 6, 1000 Ljubljana, Slovenia; urban.pavlovcic@fs.uni-lj.si; 2 Yaskawa Slovenija d.o.o., Lepovče 23, 1310 Ribnica, Slovenia; Peter.Arko@yaskawa.eu.com

**Keywords:** 3D-scanner calibration, laser scanning, hand–eye calibration, laser triangulation profilometer, robotic vision

## Abstract

A method for simultaneous laser profilometer and hand–eye calibration in relation to an industrial robot as well as its implementation is presented. In contrast to other methods, the new calibration procedure requires the measurement of only one reference geometry to calculate all the transformation parameters. The reference geometry is measured with a laser profilometer from 15 different poses. The intrinsic parameters of the profilometer, as well as the extrinsic (hand–eye) parameters, are then numerically optimized to achieve the minimum deviation between the reference and the measured geometry. The method was characterized with experiments that revealed a standard deviation of the displacements between the reference geometry after the calibration of less than 0.105 mm in the case of using the robot-arm actuator and 0.046 mm in case of using a 5-axis CNC milling machine. The entire procedure, including measurement and calculation, can be completely automated and lasts less than 10 min. This opens up possibilities for regular on-site recalibration of the entire system.

## 1. Introduction

Laser profilometers enable rapid and accurate three-dimensional (3D) measurements of complex surfaces. Their simple principle of measurement, based on laser triangulation [1], offers a compact and robust design, enabling their wide application in many areas of product quality control [2], reverse engineering [3] and adaptive machinings, such as milling [4], deburring [5] and welding [6,7,8].

Since these profilometers measure a single profile of the cross-section between the laser plane and the measured surface, a scanning movement must be used to acquire the complete 3D shape of the inspected object. The simplest designs use single-axis translational or rotational actuation, which makes it possible to capture the surface of simple geometry. On the other hand, multi-axis manipulators, such as coordinate measuring machines [9] and industrial robots [10,11], are used in situations with more complex geometries.

A major advantage of robots is their positioning flexibility, which enables automated measurements from different poses so as to avoid shading problems, maintaining the right working distance and achieving uniform sampling. The laser profilometer is attached to the end of the robot arm, which can move it in all six degrees of freedom. To transform the acquired profiles in a global coordinate system, an accurate position and orientation of the profilometer relative to the robot’s last joint must be determined, which involves a so-called hand–eye calibration [12,13] (also called robot–sensor calibration or system calibration). Furthermore, the profilometer itself must also be calibrated to remove the nonlinearities of optical distortion, camera and laser projector misalignments and the triangulation principle itself.

There have been many different calibration approaches to the robot–laser scanning system [11,12,14,15]. These approaches are usually composed of two separate steps: (i) profilometer and (ii) hand–eye calibration [11,16]. In studies where the authors used a commercial profilometer, they rely on the manufacturer’s sensor calibration and focus only on the hand–eye part of the calibration [13]. However, in the industrial environment, a sensor must be recalibrated regularly or in the occurrence of any exceptional event such as replacement of optical parts (protective window, camera’s lens, laser line projector) and collision. Even during the simplest procedures, 3D profilometers can be disassembled and reinstalled on the robotic arm, meaning the calibration data are no longer valid. Hence, the need for a procedure where the intrinsic parameters, as well as hand–eye parameters extrinsic, can be determined on the site is obvious.

The hand–eye calibration often mimics the well-known method of the tool center point (TCP) determination [17] by touching a fixed spike with an end-effector from different orientations. In [13,15], the authors measured a sphere using a profilometer in various orientations and determined its center instead of physically touching the spike. However, these methods do not calibrate the intrinsic parameters of the laser profilometer, which must be calibrated separately.

The established approach of an intrinsic calibration is based on measuring a reference 2D plate with a circle grid [14], holes [11] or checkerboard [18] that is acquired in various poses. However, the critical step is accurate detection of the reference grid, which is distorted due to an oblique projection and the camera’s lens [14]. To improve the detection accuracy, additional, uniform illumination is proposed [11]. Anyway, these approaches demand different data processing as during normal measuring regime to detect reference markers, which can introduce additional measurement uncertainty. Similarly, systematic offsets may be introduced during additional illumination due to chromatic aberrations. Our concerns also lie in the applicability to the industrial environment, where simplicity and robustness are of paramount importance.

Some authors calibrated multiple aspects of the system in a single step. For example, in [19] and [20], the kinematic parameters of a robot and the hand–eye parameters were calibrated simultaneously. Authors in [21] used the same set of calibration images for the profilometer and hand–eye calibration, but the procedure itself was still composed of several distinct steps. No reports were found on a simultaneous calibration of the hand–eye and intrinsic parameters of the profilometer.

To skip the detection of features on the reference plate, authors in [22,23] introduced the calibration of laser-scanning apparatus based on measuring a reference 3D geometry with a series of perpendicular grooves. The intrinsic and extrinsic parameters are then numerically optimized by minimizing the average deviation between the measured and the reference geometry. This approach allows the use of the same operating parameters, both during the scanning of the reference geometry and during normal operation, which prevents possible detection offsets. Moreover further, the reference plate can be an integral component of the measuring system that is periodically used for accuracy inspections. However, the 3D sensor was not attached to the robot arm or other type of actuator; thus, hand–eye parameters were not calculated. On the other hand, the rail track inspection system [24] based on laser profilometry and a 2D laser scanner attached to the portable mobile platform for mapping the outdoor environment [25] was calibrated using Gauss–Helmert model. The reference geometry composed of multiple nonparallel planes was measured in the first step, and numerical optimization of only extrinsic transformation parameters was executed in the second step. They achieved high precision results; however, the intrinsic calibration was not included.

In this paper, we present a method for the simultaneous hand–eye and intrinsic calibration of the laser profilometer, which is attached to the robot arm. The method is based on measuring the reference geometry from various positions and orientations using the same robot. The measured 3D data are then used to numerically optimize the transformation parameters in order to achieve the minimum deviation between the measured and the reference geometry. The geometry of the latter is known with an uncertainty of an order of magnitude lower than the expected deviations. Hence, our intention was to develop a procedure, which can be performed in an industrial environment, at the end customer, with as little additional equipment and operator intervention as possible. In addition, the calibration should be performed on the data that is captured and post-processed in the same way as the production measurements. This means a single-step procedure, no feature extraction, external positioning devices, additional light sources, etc., only a simple, robust reference plate.

The paper is divided into six sections. The experimental system is described first. Then the mathematical model of the profile transformation into the chosen coordinate system is presented, together with all the intrinsic and extrinsic parameters. The Section 4 describes the calibration procedure, which is divided into the measurement part, the assessment of the initial values of all the transformation parameters, their numerical optimization and software implementation. The Section 5 presents the characterizations of the method from different aspects such as convergence and measurement uncertainty according to variation of important procedure parameters. The conclusions are drawn in the Section 6 to summarize the study.

## 2. Experimental System

**Figure 1:**
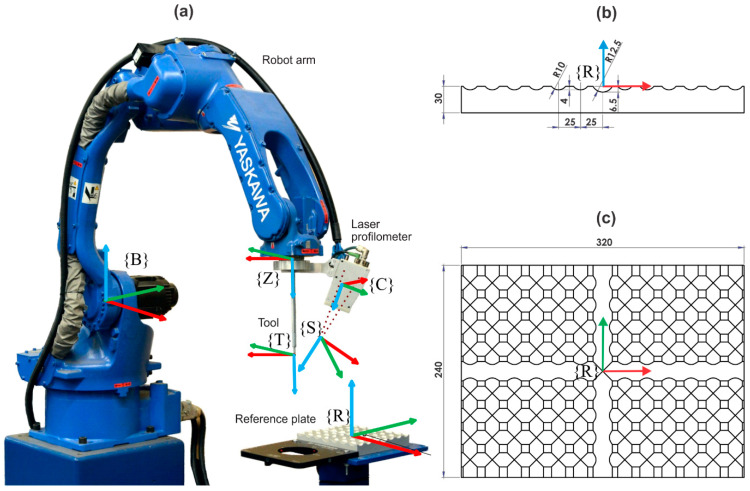
(**a**) The experimental system for the calibration procedure, (**b**) side view of the reference plate and (**c**) top view of the reference plate. The coordinate systems (cs) are realized by robot base cs {B}, robot zero cs {Z}, sensor cs {S}, camera cs {C}, tool cs {T}, and cs of the reference plate {R}.

The system is composed of three main components (see Figure 1): laser profilometer (Yaskawa MOTOSense, Ribnica, Slovenia), six-axis industrial robot (Yaskawa MA1440, Fukuoka, Japan) with the corresponding controller (Yaskawa DX 200, Fukuoka, Japan), and a reference plate that is used for hand–eye and intrinsic calibration of the system after initial assembly and for periodic system recalibration. The laser profilometer itself has an approximate measurement range from 35 mm to 75 mm at a distance between 50 mm and 150 mm from the sensor. At the maximum distance, the precision is 0.1 mm in the vertical and lateral directions [26].

A camera (manufacturer Basler, Ahrensburg, Germany, model Ace acA640-120, resolution 659 × 494 pixels, 120 frames per second) inside the laser profilometer captures images of the surface illuminated by a laser line projector (manufacturer Laser Components, Olching, Germany, model Flexpoint MVnano, wavelength 660 nm, line thickness approx. 100 µm at the focus distance), which is also a part of the profilometer. Simultaneously with each image, a robot’s pose is acquired from the robot controller, and both are fed into an industrial computer (Intel Core i7-7700HQ, 2.80 GHz processor and 16 GB of RAM). A custom-developed LabView-based software (manufacturer National Instruments, Austin, USA) communicates with the camera and the robot controller and processes the acquired data to detect the profiles with a subpixel resolution from each image and further transform them into a 3D space of the reference coordinate.

Several coordinate systems (cs) are used in the subsequent description of the system and are presented in Figure 1, where red, green and blue arrows are used for X, Y and Z axes, respectively. The robot base cs {B} is located above the motor of the first turning axis, defined by the manufacturer during the robot’s calibration. The zero cs {Z} is located in the center of the flange of the last axis. The sensor cs {S} is located at the intersection of the laser plane and the camera’s optical axis. The camera’s cs {C} is attached to the principal point of the camera’s lens of the laser profilometer. The tool cs {T} is located at the working point of the attached tool. Moreover, the cs of the reference plate {R} is attached to the center of the reference geometry.

The latter is shown in Figure 1b,c. It is milled from an aluminum plate, 320 mm long, 240 mm wide and 30 mm thick, with perpendicular round channels. Two central channels are 6.5 mm deep and have a radius of 12.5 mm, whereas all the other channels are 4 mm deep and have a radius of 10 mm. The distances between channels are 25 mm. Axes X_R_ and Y are collinear with the central channels, and the Z-axis is directed out of the plate. Its shape has an accuracy of 0.01 mm in the vertical and horizontal direction according to the CNC milling machine’s specification. The surface was sandblasted after the milling to ensure diffuse reflection of the laser light.

The geometry was designed to ensure a change of the deviation between the measured and the reference geometry after the variation of any transformation parameter (see Section 4). Only that way optimization of the transformation parameters can converge to a stable solution.

## 3. Transformation Model

**Figure 2:**
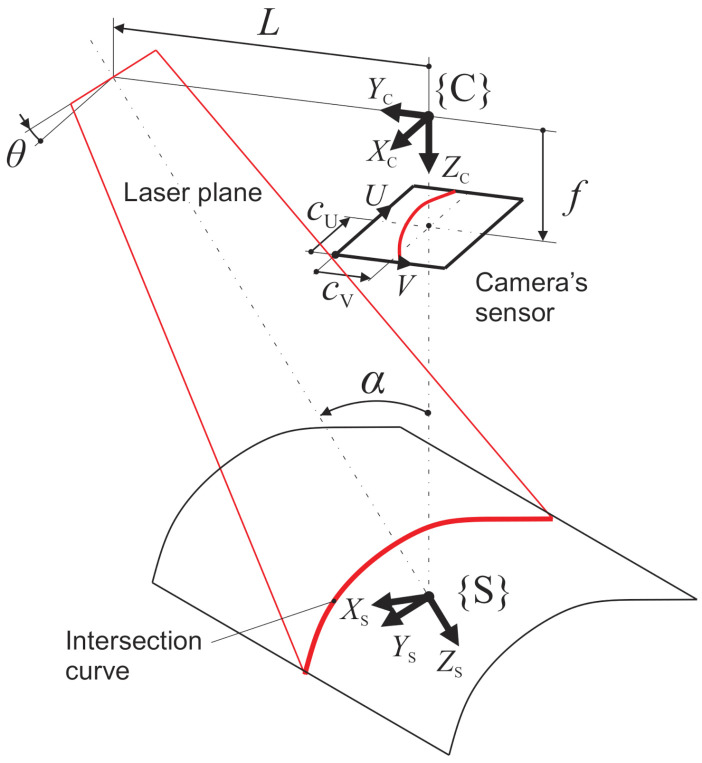
Sketch of the arrangement of the camera and the laser projector with their corresponding coordinate systems.

The laser profilometer is based on the triangulation principle (see Figure 2), where a laser projector projects a laser plane towards a measured surface. An intersection curve is formed at the intersection of the laser plane and the measured surface, which is observed by a camera from a different viewpoint.

Once the camera captures an image of the illuminated surface, the location of the profile, which is an image of the intersection curve, is detected in each column (V direction) of the image with sub-pixel resolution [27]. This means that profile location along V direction is detected with a precision better than 0.1 pixels. That leads to the coordinates *u* (index of the column) and *v* (height within the column) of the intersection profile. After this, the following model is used to transform each point of the profile into the respective coordinate system (e.g., cs of reference geometry).

Lens distortion is corrected in the first step using the Brown–Conrady model [28]. The original coordinates *u* and *v* are transformed to undistorted coordinates of the detected intersection curve *u*_UD_ and *v*_UD_:

(1) $$u_{\mathrm{UD}}=u+\left(u-c_{U}\right)\left(k_{1}r^{2}+k_{2}r^{4}\right)+p_{1}\left(r^{2}+2{\left(u-c_{U}\right)}^{2}\right)+2p_{2}\left(u-c_{U}\right)\left(v-c_{V}\right)$$

(2) $$v_{\mathrm{UD}}=v+\left(v-c_{V}\right)\left(k_{1}r^{2}+k_{2}r^{4}\right)+2p_{1}\left(u-c_{U}\right)\left(v-c_{V}\right)+p_{2}\left(r^{2}+2{\left(v-c_{V}\right)}^{2}\right)$$

where $r^{2}={\left(u-c_{U}\right)}^{2}+{\left(v-c_{V}\right)}^{2}$*c*_U_, *c*_V_ are coordinates of the principal point [9] on the camera’s sensor, *k*_1_, *k*_2_ are radial distortion coefficients and *p*_1_, *p*_2_ are tangential distortion coefficients. The normalized direction vectors *u*_N_ and *v*_N_ are then calculated as [22]:

(3) $$u_{N}=\frac{\left(c_{U}-u_{\mathrm{UD}}\right)\cdot d_{U}}{f}$$

(4) $$v_{N}=\frac{\left(c_{V}-v_{\mathrm{UD}}\right)\cdot d_{V}}{f}$$

where *f* is the focal length of the camera lens, and *d*_U_, *d*_V_ are the pixel dimensions. A profile points in the camera’s coordinate system {C} $P_{C}={\left[\begin{array}{cccc} X_{C} & Y_{C} & Z_{C} & 1 \end{array}\right]}^{T}$ are then calculated using the model described in [22]:

(5) $$Z_{C}=\frac{L}{v_{N}+\tan\left(\alpha \right)}$$

(6) $$X_{C}=Z_{C}\cdot v_{N}$$

(7) $$Y_{C}=-Z_{C}\cdot u_{N}$$

where *L* is a baseline, i.e., the distance between the projector and the camera in the *Y*_C_ direction and *α* is a triangulation angle between the projector’s and the camera’s optical axes. The points are then transformed from {C} to {S} using the following Equation:

(8) $$P_{S}=RT_{C\text{-}S}\cdot P_{C}$$

where $P_{C}$ and $P_{S}$ are point vectors and **RT**_C-S_ is a homogeneous transformation matrix from {C} to {S}:

(9) $$RT_{C\text{-}S}=\left[\begin{array}{cccc} \cos\left(\theta \right)\cos\left(\alpha \right) & -\sin\left(\theta \right) & \cos\left(\theta \right)\sin\left(\alpha \right) & 0\\ \sin\left(\theta \right)\cos\left(\alpha \right) & \cos\left(\alpha \right) & \sin\left(\theta \right)\sin\left(\alpha \right) & 0\\ -\sin\left(\alpha \right) & 0 & \cos\left(\alpha \right) & \frac{Z_{C}\cdot L}{\tan\left(\alpha \right)}\\ 0 & 0 & 0 & 1 \end{array}\right]$$

where *θ* is an angle between the laser plane and the *Y*_S_ axis. The *Z*_S_ axis is coincident with the *X*c*Z*c plane. Therefore, only two angles (*α* and *θ*) are needed to completely define the orientation of the {S}. The points are further transformed from {S} to {Z} and then into {B}, where the profilometer movement by a robot arm is considered:

(10) $$P_{B}=RT_{Z\text{-}B}\cdot RT_{S\text{-}Z}\cdot P_{S}$$

where **RT**_Z-B_ and **RT**_S-Z_ are homogenous transformation matrices for transformations from {Z} to {B} and from {S} to {Z}, respectively. Information about the **RT**_Z-B_ is provided by the robot controller that streams the data to the system computer in real time, simultaneously with each recorded image, as described in [29]. Meanwhile, the **RT**_S-Z_ is dependent on the position of the laser profilometer relative to the {Z} and is calculated during the hand–eye calibration.

Finally, the transformation into the cs of the reference geometry {R} is made to calculate a deviation between measured and reference geometry:

(11) $$P_{R}=RT_{B\text{-}R}\cdot P_{B}$$

where **RT**_B-R_, is homogenous transformation matrix for transformation from {B} to {R}. This transformation is used only during calibration, while in normal operation, it is omitted or replaced with transformation into a user-defined coordinate system.

As can be seen from the presented transformation model, Equations (1) to (9) represent point transformations inside the profilometer where 12 intrinsic transformation parameters must be known. Furthermore, in Equation (10), six additional parameters are defining **RT**_S-Z_, so-called hand–eye: three translations X_S-Z_, Y_S-Z_ and Z_S-Z_ and three rotations RX_S-Z_, RY_S-Z_ and RZ_S-Z_. Moreover, finally, in Equation (11), the matrix **RT**_B-R_ is similarly defined by three translations X_B-R_, Y_B-R_ and Z_B-R_ and three rotations RX_B-R_, RY_B-R_ and RZ_B-R_. Thus, 24 transformation parameters should be precisely determined during the calibration procedure to achieve accurate 3D measurements.

## 4. Calibration Procedure

**Figure 3:**
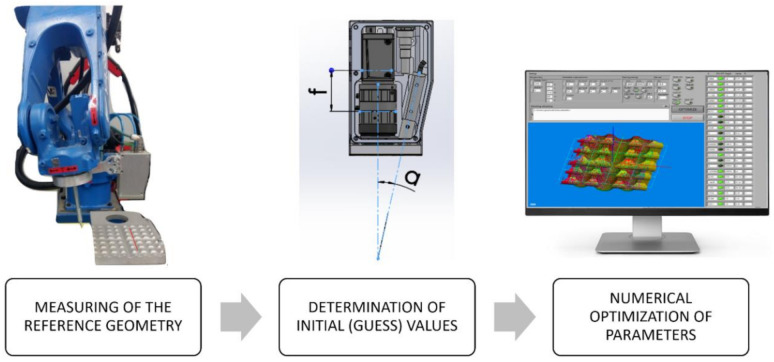
Basic steps of the calibration procedure.

The calibration procedure is schematically presented in Figure 3. It is divided into measuring of the reference geometry, determination of initial (guess) values and numerical optimization of the transformation parameters. Each step is described in more detail below.

### 4.1. Measurement of the Reference Geometry

**Figure 4:**
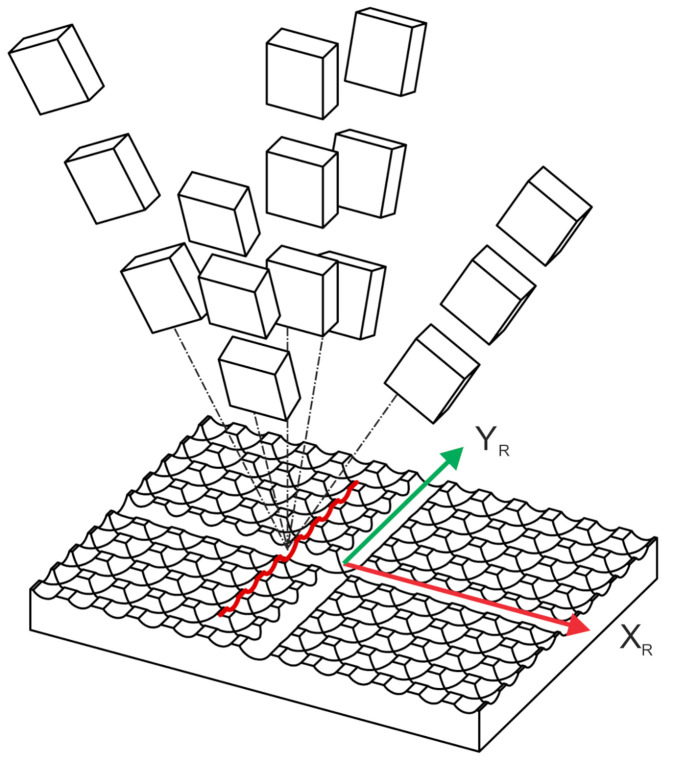
Poses of laser profilometer during the scanning of the reference plate.

In the first step, the measurements of the reference plate are acquired from 15 different poses, as shown in Figure 4, where three distances from the plate (75 mm, 100 mm and 125 mm) are combined with three rotations (−10°, 0° and 10°) around the X_R_ and (−5°, 0° and 5°) Y axes. The scanning direction is approximately parallel to the X_R_ axis in all poses.

By changing the robot orientation, we increased the sensitivity of the deviation between reference and measured plate to the hand–eye parameters. However, the angles were limited to minimize the influence of the robot positioning inaccuracy. This is vital in applications where the robot orientation must be changed during the scanning to be perpendicular to the measured surface, such as the case of 3D seam tracking [4] or geometry inspection of parts with complex shapes [30].

A smaller range of rotations around the Y-axis was chosen to prevent an excessive reduction of maximal and minimal measuring distances since we want to assure that the entire profile width was always within the sensor’s measuring range.

### 4.2. Estimation of Initial Values of Transformation Parameters

**Figure 5:**
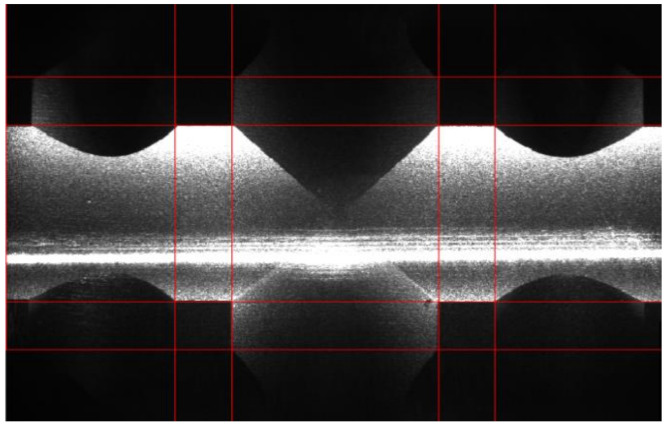
An example of a good alignment between the overlaid mesh (red lines) and the reference plate, illuminated by a laser line. The central channels are in the center of the image. The white horizontal line is an overexposed laser line since a camera’s exposure time is increased from 400 μs to 30 ms.

The initial values of the intrinsic parameters are estimated from the sensor’s geometry in the product documentation and the specifications of the camera and lens. The distortion parameters (*k*_1_, … *p*_2_) are set to zero, while the principal point (*c*_U_, *c*_V_) is set to the middle of the sensor. The initial **RT**_S-Z_ transformation (hand–eye transformation) is estimated by positioning the profilometer perpendicularly to the reference plate, which is achieved by visual observation of live images from the profilometer’s camera (see Figure 5). The proper pose is achieved when the central channels of the reference geometry overlap with the red overlay mesh. The pose of the reference geometry relative to the base cs is determined by touching the diagonal corners of the reference geometry with the robot’s working tool. We must emphasize that this procedure must be carried out only after the installation of the robot and the reference plate on the factory floor, while during periodical recalibration routines, it is not necessary.

### 4.3. Numerical Optimization

**Figure 6:**
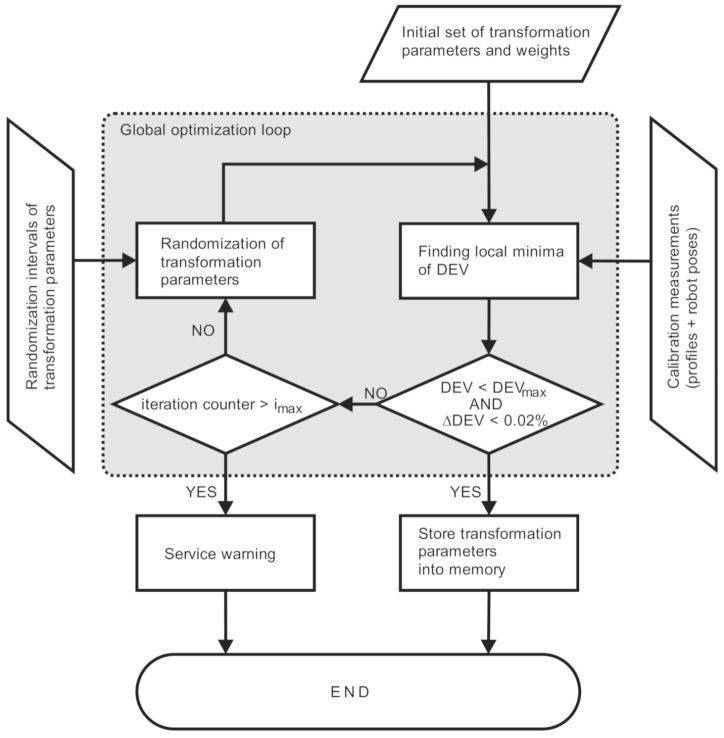
Flow chart of the calibration algorithm.

Numerical optimization of the transformation parameters starts by importing the calibration measurements into the custom-developed calibration program of the MOTOSense software (Yaskawa, Ribnica, Slovenia), which implements an algorithm described in Figure 6.

Each of the 15 calibration measurements contains a sequence of detected profiles in the image coordinate system (*u*, *v*) and the corresponding poses of the robot arm (X_Z-B_, Y_Z-B_, Z_Z-B_, RX_Z-B_, RY_Z-B_ and RZ_Z-B_). Thus, the measurements are stored as data tables of N_data_ × (6 + M_data_) rows and columns, where *N_data_* is a number of profiles, *M_data_* is the length of a profile (equal to the image width) and six values of the robot pose at the time of the image acquisition are appended to each row.

**Figure 7:**
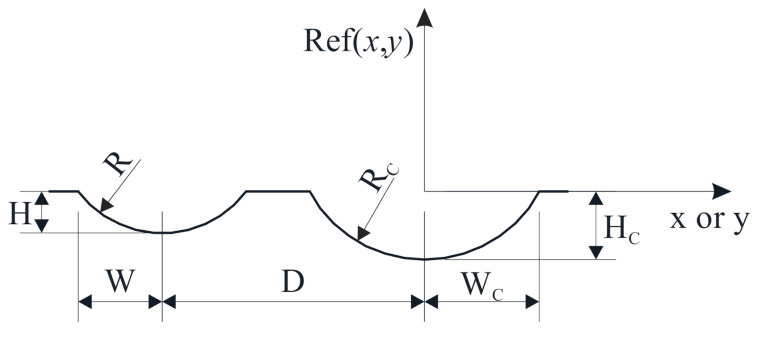
Dimensions of the reference geometry.

The initial set of the transformation parameters, corresponding weights and randomization intervals are additionally read from the datafile at the start. From these inputs, the optimization of the transformation parameters is performed using Powell’s optimization algorithm [31] for finding the local minimum of the merit function (DEV), defined as a deviation between the measured and the reference geometry:

(12) $$\mathrm{DEV}\left(C\right)=\sqrt{\frac{1}{N}\sum_{i=0}^{N-1}{\left(\mathrm{Ref}\left(X_{R}{\left(C\right)}_{i},Y_{R}{\left(C\right)}_{i}\right)-Z_{R}{\left(C\right)}_{i}\right)}^{2}}$$

where *N* is the number of measured points, **C** is the vector of the transformation parameters, *X*_R_(**C**)*_i_*, *Y*_R_(**C**)*_i_* and *Z*_R_(**C**)*_i_* are the coordinates of the *i*-th measured point in the {R}, reconstructed using the transformation model defined with Equation (1) to Equation (11). The function Ref(*x*, *y*) returns the height of the reference geometry (see Figure 7) for coordinates *x* and *y*, which is defined as follows:

If (|*x*| < W_C_ or |*y*| < W_C_), then:

(13) $$\mathrm{Ref}(x,y)=R_{C}-H_{C}-\sqrt{R_{C}{}^{2}-\min\left(x^{2},y^{2}\right)}$$

If (|*x*| mod D) < W or (|*y*| mod D) < W), then:

(14) $$\mathrm{Ref}(x,y)=R-H-\sqrt{R^{2}-\min\left({\left(\left|x\right|\mathrm{mod}D\right)}^{2},{\left(\left|y\right|\mathrm{mod}D\right)}^{2}\right)}$$

Else if none of above conditions is true, Ref(*x*,*y*) = 0.

Dimensions of the grooves are presented in Figure 7, where the distance between grooves (D) is equal along *x* and *y*-direction. Equation (13) is used for points, which are within the region of central grooves, while Equation (14) is used for points, which are within any noncentral groove. If the point is within the region where the central grove is crossing with anyone of a noncentral one, then the Ref(*x*, *y*) returns the minimum of Equations (13) and (14).

An integral part of Powell’s optimization algorithm is so-called bracketing [32] that brackets the minimum of DEV along the optimization direction. To assure proper functioning of this subroutine, the initial guess of brackets must generate approximately the same changes of DEV for any combination of transformation parameters, which is done by a normalization of the transformation parameters with weights:

(15) $$P_{j}=C_{j}/Weight_{j}$$

where **C**_j_ is the j-th transformation parameter and **Weight**_j_ is the corresponding factor used to equalize the sensitivity of DEV if any parameter **P**_j_ is changed for the same step. Typical values of the **Weight** are listed in Table A1 in Appendix A. A higher value is used for those parameters that cause a smaller change in the DEV value. Such are the hand–eye rotations and intrinsic parameters, in particular distortion parameters.

To find the global minimum of the DEV, the vector of optimal parameters $C^{\mathrm{opt}}$ is randomized after finding the local minima:

(16) $$C^{\mathrm{new}}{}_{j}=C^{\mathrm{opt}}{}_{j}+\mathrm{rand}(-RI_{j},RI_{j})$$

where function rand returns a random number of uniform probability within the interval ±**RI**_j_. Typical values of randomization intervals are listed in Table A1 in Appendix A.

If the newly found DEV is lower than the one in the previous iteration, the current parameters are stored as the new optimum ($C^{\mathrm{opt}}$), otherwise the previous remains. This enables jumping between neighbor minima in a downhill direction, but the downside of this is significantly longer calculation time due to many futile iterations, where the new set $C^{\mathrm{new}}$ is either in close vicinity of the current optimum or too far and in the wrong direction. The same optimum is found in the first case and less optimal in the second one.

The iteration stops when the relative change of DEV is lower than the prescribed tolerance (typically ΔDEV < 0.02%) and its value is lower than the declared threshold (typically DEV_max_ = 0.11 mm). The decrease of DEV is therefore 24 nm at the convergence criterion, or less than 3‰, according to the accuracy of the reference geometry. Then the transformation parameters are stored in the system memory. Otherwise, if convergence is not reached after the maximum number of iterations, a service warning is messaged to check the robot’s accuracy, clean the optics, set the image-acquisition parameters, or refocus the optics of the camera and projector.

### 4.4. Software Implementation

A graphical user interface (GUI), a data reading and storage are programed in LabView 2015, while the optimization kernel is implemented as a dynamically linked library (dll) in C++, using the library Numerical Recipes in C [32].

**Figure 8:**
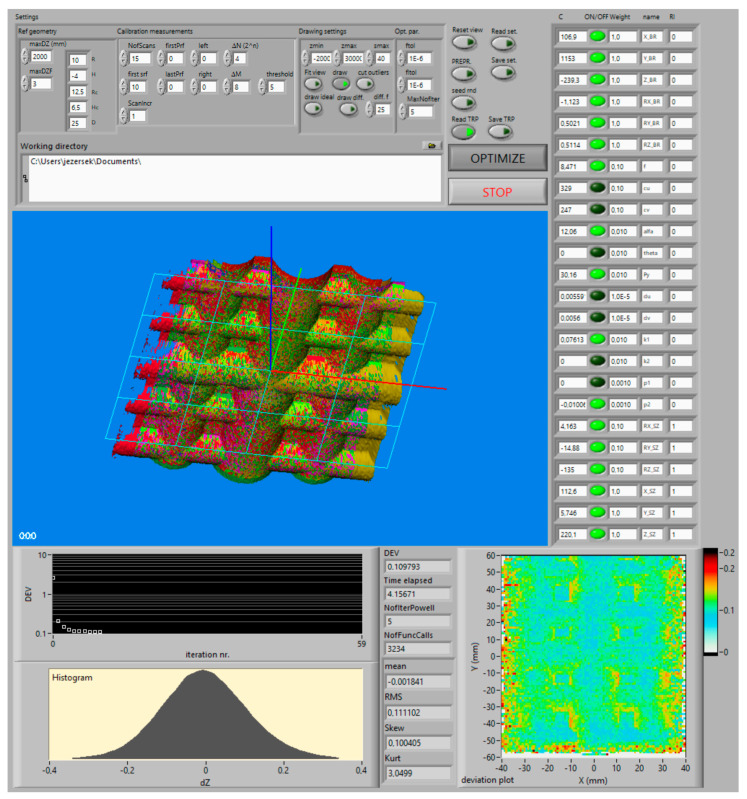
Graphical user interface of the calibration program.

Figure 8 shows the GUI, which consists of Settings (upper part), a 3D display of the measured geometry and indicators for numerical and graphical presentation of current goodness of fit between measured and reference geometry. A very important part is a table of transformation parameters (C) together with switches for including/excluding them into optimization (on/off), their names, weights for normalization and randomization intervals (**RI**). These controls enable us to study the influences of various transformation parameters on the final result.

Settings related to the reference geometry define its dimensions (R, H, R_C_, H_C_ and D). Moreover, two thresholds (maxDZ and maxDZF) are used to control the rejection of outliers from calibration measurements. The first represents the absolute value, and the second a multiplication factor of the current DEV value. Thus, the i-th point is considered in the calculation of the next DEV if the following condition is true:

(17) $$\left|\mathrm{Ref}\left(X_{R}{\left(C\right)}_{i},Y_{R}{\left(C\right)}_{i}\right)-Z_{R}{\left(C\right)}_{i}\right|<\min\left(\mathrm{maxDZ},{\mathrm{DEV}}^{\mathrm{opt}}\cdot \mathrm{maxDZF}\right)$$

In settings related to the calibration, measurements are parameters that control the number of scans, their clipping and point-cloud reduction in order to speed-up the calculation and to remove part of scans that do not cover the reference geometry. The clipping is done by deleting the scanned points outside of the boundary offset defined by (firstPrf, lastPrf, left, and right), while the point-cloud reduction is done by extracting every *ΔN* × *ΔM* point from the scanning data, where *ΔN* is a reduction factor along profile length, and *ΔM* is a reduction factor along the scanning path.

Parameters inside the drawing settings control the 3D display to draw either measured or reference geometry or differences between them along Z-direction. The last option is especially useful for checking the presence of any systematic deviations.

Similar monitoring offers a deviation plot, where colors represent local average deviation mapped with a resolution of 1 × 1 mm. Moreover, finally, the deviation is also presented as a histogram of differences, where we can check if its distribution is similar to the Gaussian. This is the second in-depth check to see how many systematic effects are still noticeable above the level of noise in the current result and can be corrected with the calibration of the selected transformation parameters. However, the deviation plot cannot indicate the systematic effects which are related to the inaccuracy of the reference geometry or the robot pose.

## 5. Characterization of the Calibration Method

The developed calibration method was characterized with several tests in order to point out the parameters which have an important influence on the convergence of DEV function and uncertainties of optimized transformation parameters (3D transformation uncertainty). We first tested the influence of the chosen set of optimized parameters and the associated complexity of the transformation model on the achieved deviation between the measured and reference geometry. The influence of measured point-cloud density was further analyzed to choose a compromise between the calibration uncertainty and a processing time. The calibration uncertainty was tested by repeated measurements after various changes of the laser profilometer pose against the robot (hand–eye parameters). Further, the combined standard uncertainty of the entire system was assessed based on Guide to the expression of uncertainty in measurement (GUM) [33]. Finally, we compared the results of the same calibration procedure where in the first part, the robot was used as an actuator and, in the second part, a highly accurate 5-axis computer CNC milling machine. These tests gave us additional insight into the influence of the positioning accuracy of the robot actuator on the overall measurement uncertainty.

The room temperature was 21 °C during all experiments, and the system was started at least two hours before the measurements so that all subsystems were thermally stabilized. In all tests, each scanning measurement consists of 15 separate surface measurements (scans), where each contains 426 profiles with a resolution of 0.28 mm along the X-axis (scanning axis). The time needed for a reference plate measurement from all 15 positions is about 280 s, where the measurement from every position is 120 mm long and consists of about 273,000 points. The same computer was used for numerical calculation as in the experimental system (see Section 2).

### 5.1. Selection of Optimized Transformation Parameters

**Figure 9:**
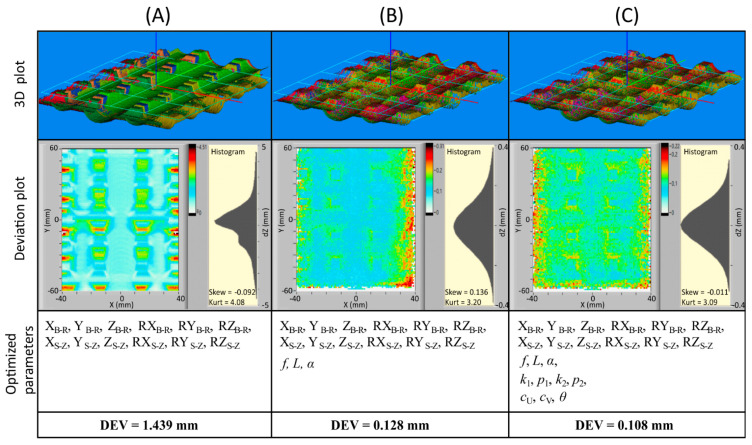
Calibration results for different sets of transformation parameters being optimized. In (**A**), only extrinsic parameters were optimized. Focal length *f*, projector distance *L* and triangulation angle *α* were added in (**B**), and all parameters were included in (**C**). 3D measurements of the reference plate are shown in the first row, where colors represent measurements from various poses. The second row shows deviation plots and histograms of differences between the reference and measurement geometry.

Figure 9 shows the calibration results for different sets of optimized transformation parameters, where 3D plots of the measured geometry are shown in the first row and deviation plots in the second row together with histograms of differences between the reference and measured geometry. Transformation parameters that were optimized are listed in the third row and resulted in DEV value after optimization is displayed in the last row.

In the first set (column A), only extrinsic parameters (hand–eye **RT**_S-Z_ and base-to-reference **RT**_B-R_) were optimized, while all other parameters were fixed at the initial values (see Table A1). A large lateral deviation along Y-axis (approximately 15 mm) between individual measurements can be seen from the 3D plot. Due to groove-shaped reference geometry, these deviations also result in the vertical direction, which is visible on the deviation plot with maximal amplitude near 4.5 mm. It should be noted that such conversion between horizontal and vertical deviations appears only in regions where the reference geometry is sloped.

Deviation plots are together with histograms, convenient visual indicators of goodness of the fit between the measured and the reference geometry. This means that any systematic offset will be visible as a consistent color variation on a deviation plot and also as a deviation from Gaussian distribution on a histogram (skew ≠ 0 and kurt ≠ 3). For example, if we look at the second case (column B), where three basic intrinsic parameters (focal length, triangulation angle, and baseline) were added to the optimization set, we can conclude from the DEV value (0.128 mm) that the system is finely calibrated. Nevertheless, the deviation plot reveals some systematic deviations on the right edge of the region, and also, the edges of the grooves are still clearly visible in the central region of the plot. The histogram also shows some skewness (0.136) and kurtosis (3.20) of the distribution, which furthermore proves that additional transformation parameters should be included in optimization.

The third set (column C) shows the results, where all parameters (hand–eye and intrinsic) were optimized. In this case, the systematic deviations are nearly invisible on the deviation plot, and the distribution is practically Gaussian (skew = −0.011 and kurt = 3.09). These two indicators prove that optimization found a good minimum with an average deviation DEV = 0.108 mm.

The optimization with an entire set of parameters was run 8-times, where the initial values of hand–eye parameters were randomly varied by ±5° and ±7.5 mm in terms of rotation and translation, respectively. This variation was done to calculate the measurement uncertainties of each parameter by calculating a standard deviation. The average value and standard deviation of each parameter are listed in the third and fourth columns in Table 1. We can see that majority of parameters have small standard deviations; however, some of them have extremely high relative deviations (marked with **). For example, the relative deviation of *c*_U_ is higher than 5% and of *θ* higher than 280 % However, the overall uncertainty of the DEV value is still very small (0.00022 mm).

By observing the changes of parameters during optimization, we found that some of the parameters with high standard deviation values are correlated. This means that a change of one parameter is compensated by changing one or more other parameters resulting in an almost constant DEV function. This appears as a slow but constant sliding of their values during optimization. A typical example of such interdependent parameters is image center location (*c*_U_ and *c*_V_) and translational part of hand–eye parameters (X_S-Z_, Y_S-Z_ and Z_S-Z_). Yet another group consists of a rotation of laser projector (*θ*) and rotational part of hand–eye parameters (RX_S-Z_, RY_S-Z_ and RZ_S-Z_).

Therefore, in the next step, we fixed *c*_U_, *c*_V_ and *θ* to the initial values. After the repeated procedure of multiple optimizations, we found that the deviation between the reference and measured geometry remained the same (DEV = 0.1081 mm) while deviations of hand–eye parameters were significantly reduced. These results are listed in the fifth and sixth columns of Table 1.

After fixing the mentioned parameters, we found relatively large deviations only in the distortion parameters (*k*_1_, *k*_2_, *p*_1_ and *p*_2_). Therefore, the parameters *k*_2_ and *p*_2_ were fixed to 0 values in the third scenario without significantly deteriorating the value of DEV, which is presented in the last two columns of Table 1.

However, we must emphasize that fixation of certain parameters also depends on the specific configuration of a laser profilometer, for example, the magnitude of optical distortions, which affects the degree of the measuring system nonlinearity. The Gauss–Helmert model with constraints [24,34] is one of the possible improvements of the optimization algorithm.

### 5.2. Influence of Point Cloud Density

The size of the original point-cloud of the reference measurement is approximately 4.1 million points due to high measuring resolution in the lateral direction (659 pixels × 426 profiles) and multiple scans of the reference plate (15 scans). All these points are transformed and compared with the reference geometry during a single calculation of REF, which is called more than 10,000 times per optimization. Since transformations and comparison of each point are process-demanding operations, it makes sense to investigate the influence of the original point-cloud reduction on the measurement uncertainty of transformation parameters, as the computational time can be significantly shortened with that. The reduced point-cloud was generated by extracting every *ΔN* × *ΔM* point from the scanning data, where *ΔN* is a reduction factor along profile length, and *ΔM* is a reduction factor along the scanning path.

**Figure 10:**
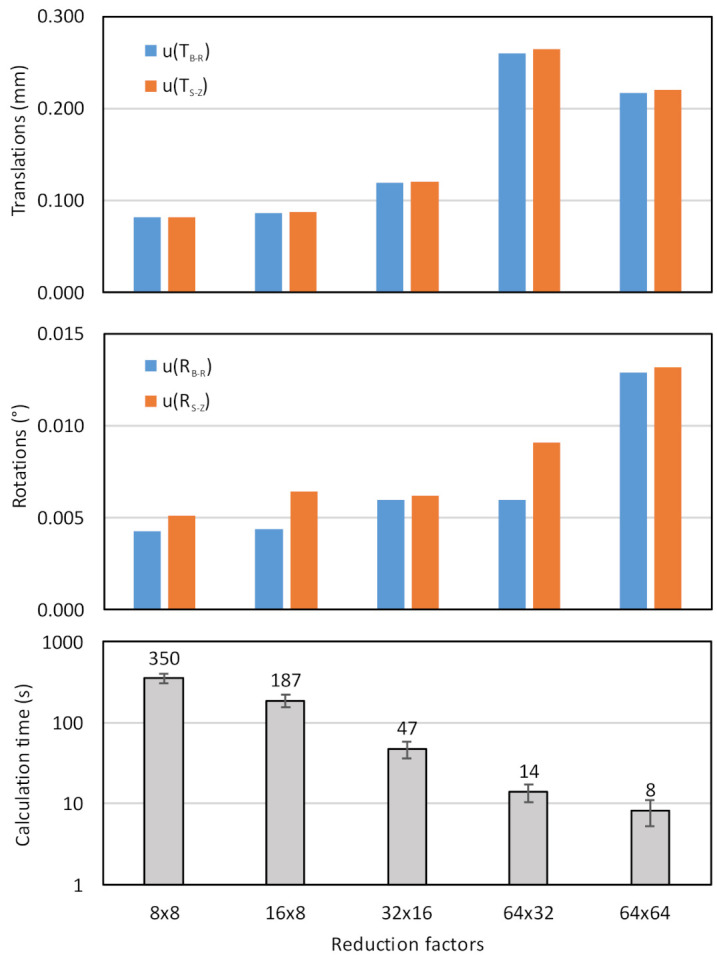
Influence of point-cloud reduction factors on measured uncertainty of extrinsic translations (upper diagram) and rotations (middle diagram) and on calculation time (bottom diagram).

Figure 10 shows the influence of point-cloud reduction size on the measured uncertainties of extrinsic parameters. The uncertainty is calculated as a Euclidean distance of standard deviations of all three translations (top diagram) and rotations (middle diagram), where eight repetitions with different initial values of transformation parameters were used. Blue bars show uncertainties of base to reference (B-R) transformation, while the red bars the sensor to zero cs (S-Z) transformations.

We can see that relatively big reduction factors (e.g., 8 × 8 and 16 × 8) do not significantly deteriorate the measuring uncertainty neither in terms of translations nor rotations. However, in these cases, the processing time is relatively long (350 s and 187 s). On the other side, if reduction factors are bigger (e.g., 64 × 32 and higher), and consequently the point-cloud size is smaller, the measuring uncertainty of translations starts increasing over 0.2 mm, which is more than twice the lowest achievable value, but the processing time decrease below 20 s.

According to the presented results, we decided to use 16 × 8 reduction factors in all further experiments since the increase of measuring uncertainties is within the acceptable limits, and the processing time is reasonably short (200 s).

### 5.3. Changes of a Hand-Eye Configuration by a Repositioning of the Laser Profilometer

The results in the previous two subsections are based on one set of measurements and variations of the optimization procedure to characterize its behavior without external disturbances. In this subsection, we analyze whether a physical change of the measuring system affects the calibration result in any way. Therefore, we performed five sets of scanning measurements of the reference plate where each time, only the pose of the laser profilometer relative to the robot was changed, thus the hand–eye parameters. Everything else, the pose of the reference plate and intrinsic parameters of the laser profilometer, remained unchanged, and we expect that they will stay constant after each calculation.

Our expectations are confirmed in Table 2. We can see that the uncertainty of the reference plate position along the X- and Y-direction is better than 0.05 mm, and it is less than 0.12 mm along Z-direction. Higher uncertainty along Z-direction is related to the relatively small rotation ranges of the laser profilometer around X_R_ and Y axes during scanning of the reference geometry. However, it is still evident, also by looking at B-R rotations, that convergence of these parameters is very good. Similarly, we can notice excellent results of all intrinsic parameters. The standard deviations are less than 0.018 mm for the focal length (*f*), less than 0.008° for triangulation angle (α) and less than 0.04 mm for the camera to laser projector (L). Standard deviations of the last six parameters in Table 2 (hand–eye parameters) are obviously higher since we intentionally repositioned the laser profilometer before each measurement.

**Figure 11:**
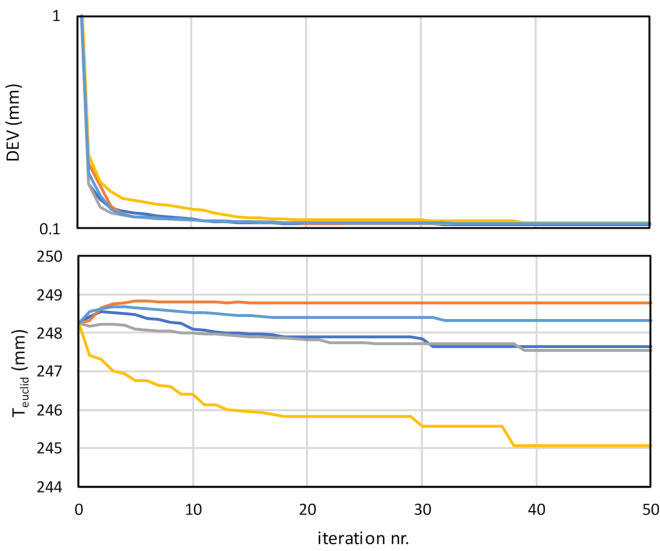
Convergence plot of the merit function (DEV) value and Euclidean distance of hand–eye translations (T_euclid_) during numerical optimization of the transformation parameters. Five curves represent repetitions of calibration measurements with different hand–eye configuration.

The convergence of the DEV and Euclidean distances of extrinsic parameters (T_euclid_) for all five measuring repetitions are shown in Figure 11. Since the initial values of the extrinsic parameters vary among the different measurements, also the initial DEV value ranges between 2 mm and 6 mm. However, in all cases, at the end of optimization, it converges below 0.11 mm. The convergence is rapid at the beginning, while it slows down after a rough approximation of the extrinsic parameters. These parameters are calculated to the values within 15% of the final values after the first 10 iterations in most cases. After this, the errors of the intrinsic parameters have a major impact on the deviations. The jumps in some curves appear when the global optimization mechanism finds a better local minimum. Due to the same reason, the convergence among different measurements may differ by more than 50% in terms of the required number of iterations to achieve the convergence criterion (DEV_max_ < 0.11 mm). For the sake of illustration of the optimization algorithm, graphs are shown in all cases up to the 50th iteration when the convergence is achieved in all cases.

The jumps between local neighbor minima are even more evident from the third diagram in Figure 11, where convergences of translations (T_euclid_) are shown. The initial value is the same in all measurements, which then converge to the value, which best describes the position and orientation of the profilometer against the robot.

Further, we analyzed the position-measuring (PM) uncertainty where we used a second object with easily extractable features to avoid the introduction of additional measuring uncertainty related to the feature detection. This object was positioned in another region of the robot’s workspace, rotated for approximately 90° around the Z-axis of base cs. Its shape was the same as the reference plate, and its pose against the base cs was determined with the same program as for the calibration. The selected position simulates restrictions inside a robot working space in industrial applications, where a robot is often positioned near a wall.

Table 3 shows the average values and corresponding standard deviations of the measured centers in all three dimensions (X_PM_, Y_PM_, Z_PM_). It can be observed that the scatter is approximately the same as the translation part of the measuring uncertainty of the reference plate (X_B-R_, Y_B-R_, Z_B-R_) shown in Table 2.

A single-factor analysis of variance (ANOVA) with a 95% level of confidence shows that the position-measured differences are not statistically significant (*p* > 0.99) if the initial extrinsic transformation parameters are randomly varied by ±5° and ±7.5 mm in terms of rotation and translation, respectively, which is inside the range required for the optimization software to find the optimal parameters on its own, without further operator interference. It was also found that the initial parameters inside that range have a negligible influence on the final result.

### 5.4. Assessment of

The combined standard uncertainty of the entire system (*u*_c_) is influenced by measuring uncertainties of basic measuring values and of all transformation parameters. The basic measuring values are the positions *u* and *v* of detected points along with each profile and the robot pose (**T**_Z-B_), which is streamed from the robot controller. There are also robot rotations, but due to the high accuracy of robot encoders and short distances between coordinate systems {Z} and {S}, their uncertainties can be neglected.

The uncertainties of *u* and *v* were estimated from the measured profiles of the reference geometry by calculation of root-mean-square deviation (RMSD) of their deviation on the planar region of the reference geometry near the image center. The resulted values of uncertainties are *u*_U_ = 0.58 pix, and *u*_V_ = 0.1 pix along U and V direction, respectively. The robot uncertainty is estimated according to published data of similar industrial robots within a limited working range [35,36]. If we take into account that less than 100 × 100 × 100 mm of translational and less than ±20° of rotational movement is needed, the assumption that *u*_robot_ = 0.1 mm is reasonable.

**Figure 12:**
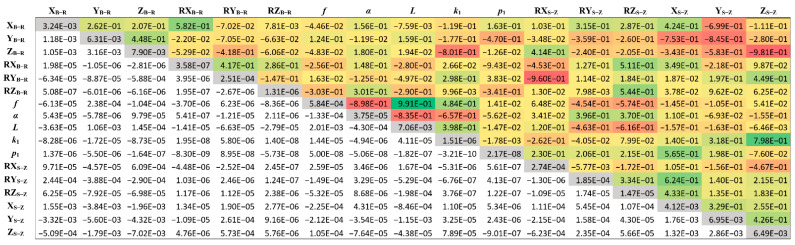
Correlation and covariance matrices. The upper triangle shows correlations and the bottom covariances. Diagonal values (gray color) are variances. Red and green colors present highly correlated parameters.

The uncertainties of all optimized transformation parameters and related covariance and correlation matrices were calculated based on repeated calibration procedures (N = 12). The measurement of the reference geometry was performed during each calibration, and the entire geometry was held constant during all repetitions. Figure 12 shows the matrix, where the upper triangular part (above the diagonal) presents correlations between the parameters, while the bottom part presents the variances.

According to GUM [33], combined standard uncertainties related to 3D transformation were calculated by using the equation for the correlated transformation parameters:

(18) $$u_{c.tp}{}^{2}=\sum_{i=1}^{N}\sum_{j=1}^{N}\frac{\partial f}{\partial C_{i}}\frac{\partial f}{\partial C_{j}}K_{C_{i}C_{j}}$$

where *N* is a number of optimized parameters, *C_i_* is *i*th transformation parameter, $\frac{\partial f}{\partial C_{i}}$ is the sensitivity coefficient of *C_i_*, and **K** is the covariance matrix (see Figure 12) of repeatedly measured transformation parameters. Sensitivity coefficients, which describe how the output measurands (X_R_, Y, and Z_R_) varies with the change of the transformation parameters, were calculated with numerical simulation of small changes in transformation parameters. Table A2 in Appendix A shows their values along all three axes.

The combined standard uncertainties, which include 3D transformation, laser profile detection and robot, are then calculated:

(19) $$u_{c{.X}_{R}}=\sqrt{u_{c.\mathrm{tp}{.X}_{R}}{}^{2}+u_{\mathrm{robot}}{}^{2}}$$

(20) $$u_{c{.Y}_{R}}=\sqrt{u_{c.\mathrm{tp}{.Y}_{R}}{}^{2}+u_{\mathrm{robot}}{}^{2}+{\left(\frac{\partial Y_{R}}{\partial c_{U}}\right)}^{2}u_{U}{}^{2}}$$

(21) $$u_{c{.Z}_{R}}=\sqrt{u_{c.\mathrm{tp}{.Z}_{R}}{}^{2}+u_{\mathrm{robot}}{}^{2}+{\left(\frac{\partial Z_{R}}{\partial c_{V}}\right)}^{2}u_{V}{}^{2}}$$

The Euclidean distance is finally used for evaluation of the scalar value of combined standard uncertainty of each measured point:

(22) $$u_{c.\mathrm{Euclid}}=\sqrt{u_{c{.X}_{R}}{}^{2}+u_{c{.Y}_{R}}{}^{2}+u_{c{.Z}_{R}}{}^{2}}$$

The propagation of the resulted standard uncertainty is summarized in Table 4, where contributions of each subsystem are listed in separate rows, while the columns present the contributions along separate directions and Euclidean distance the last column.

We can see that uncertainty of 3D transformation has more than seven times smaller contribution comparing to the profile detection. On the other hand, we can see that robot uncertainty, which is only a rough estimation, has the greatest contribution to the overall uncertainty.

### 5.5. Effect of a Robot Positioning Accuracy

In the last experiment, we replaced the robot arm with a much more accurate 5-axis CNC milling machine (Deckel Maho, Bielefeld, Germany, DMU 100 monoBLOCK iTNC 530 Heidenhain) in order to study the influence of the robot positioning uncertainty on the calibration results. We compared the average values of DEV and the position-measurement uncertainties of the second object for both systems, where the scanning measurements were acquired from the same relative positions and with the same resolution in both cases.

The comparison of the results is shown in Table 5. It is evident that the DEV is two times lower when the CNC was used. The average value of the DEV is, in this case, also two-times smaller than the standard uncertainty of the profile detection (see Table 4, *u*_c.Euclid.p.d_ = 0.085 mm), where only the uncertainty of the laser line detection is taken into account.

It is a reasonable assumption that the positioning accuracy of the CNC machine is at least an order of magnitude better than the one of the robot. Therefore, we assume that in cases of CNC, the positioning uncertainty itself is neglectable compared to the profilometers. The uncertainties in Table 5, column 5-axis CNC, are uncertainties introduced mainly by the profilometer and in the robot arm column by the robot.

Comparing with other studies, a single step calibration method of a similar system based on measuring a checkerboard was recently presented in [21]. The duration of calibration is within 10 min and the measurement accuracy within 0.40 mm. More surprising is their result of position measurement uncertainty of the second object, which is 1.855 mm.

Authors in [20] used a three-step calibration procedure, where they calibrated the robot kinematic model together with hand–eye parameters, camera and profilometer. They validated the calibration by measuring the standard sphere platform inside a measuring area of 700 mm × 500 mm × 400 mm and report an average distance error of 0.216 mm.

The exact values of robot position errors are hard to determine since they depend on a robot’s position in joint space, and the same Cartesian pose of the profilometer can be achieved by different joint poses. In our case, the distances between measuring poses are up to 100 mm, and rotation angles up to 20° on a lever of about 250 mm, so position uncertainty in a range around 0.1 mm is common for industrial robots [36]. Quantitative comparison of different calibration techniques on the same hardware system would certainly offer the best way to evaluate the pros and cons of an individual technique, as it negates the impact of different accuracies of robots and laser profilometers.

This demonstrates that for any further improvement of the robot-based 3D scanning system, we must first improve the accuracy or the robot-pose measurement. This could be achieved, for example, with an external real-time pose measurement using a laser-tracker interferometric device [37] or with more accurate robot arms, specifically designed for accuracy-demanding applications.

Further, we want to stress out that the presented method solves the hand–eye and intrinsic calibration, while the robot calibration (Denavit–Hartenberg or D-H parameters) is not part of it. Therefore, the robot pose is supposed to be accurate within the measured uncertainty obtained with separate robot calibration. However, a simultaneous robot calibration is possible using the same approach, where an additional pose measuring sensor (e.g., laser tracker) or actuator (e.g., CNC) would measure or move the reference plate in a larger area of the robot workspace.

## 6. Conclusions

A method for measuring the simultaneous hand–eye and intrinsic parameters of a laser profilometer mounted on a robot arm was developed. It is based on scanning the reference geometry with the robot from multiple poses and a numerical minimization of the deviation between the measured and the reference geometry.

The resulting average deviation was approximately 0.105 mm if the industrial robot arm was used and 0.05 mm in the case of the highly accurate 5-axis CNC actuator. The standard deviation of the measured position of the second object was similar to the calculated deviation (0.107 mm and 0.046 mm for the robot arm and CNC actuator, respectively) and shows that the calculated deviation actually relates to the measurement uncertainty of the whole system. Results of random variation of initial values of transformation parameters (±5° and ±7.5 mm in terms of rotation and translation) show excellent repeatability and robustness of the method due to wide tolerances of the initial values of parameters against their final values.

The entire procedure, including measurement and calculation, can be completely automated and lasts less than 10 min. This opens up possibilities for regular on-site recalibration of the entire system, which is particularly useful in industrial environments, where any shutdown of the production process is highly undesirable.

The results of this study suggest a further improvement of the proposed optimization method with the aim of releasing requirements of relatively precise estimation of initial values of transformation parameters. Implementation of an advanced optimization algorithm, enabling the constraints between correlated parameters, would further improve the convergence and accuracy of the calibration. Elimination of sharp edges on the reference geometry would also improve the uncertainty of the laser profile detection.

## Figures and Tables

**Table 1:** Average values of optimized transformation parameters and respective standard deviations for N = 8 repeated optimizations of the same measurement set where initial values of hand–eye parameters were randomly varied. Excessive standard deviations are marked with **, and parameters that were not optimized in the second and third scenarios are marked with *FIXED*.

		All Parameters		Without *c*_U_, *c*_V_ and *θ*		Without *c*_U_, *c*_V_, *θ*, *k*_2_ and *p*_2_	
Parameter	Unit	Average	Standard Deviation	Average	Standard Deviation	Average	Standard Deviation
X_B-R_	mm	106.91	0.004	106.91	0.004	106.91	0.005
Y_B-R_	mm	1152.89	0.014	1152.87	0.013	1152.88	0.021
Z_B-R_	mm	−239.74	0.117	−239.76	0.080	−239.73	0.084
RX_B-R_	°	−1.125	0.0003	−1.124	0.0001	−1.124	0.0002
RY_B-R_	°	0.401	0.0346	0.476	0.0046	0.477	0.0043
RZ_B-R_	°	0.505	0.0007	0.505	0.0005	0.504	0.0004
*f*	mm	8.380	0.0209	8.380	0.0127	8.386	0.0153
*c*_U_	pix	333.40	** 19.57	329.00	*FIXED*	329.00	*FIXED*
*c*_V_	pix	245.23	** 2.17	247.00	*FIXED*	247.00	*FIXED*
*α*	°	12.14	0.085	12.08	0.005	12.08	0.006
*θ*	°	−0.051	** 0.147	0.000	*FIXED*	0.000	*FIXED*
*L*	mm	29.83	0.047	29.83	0.046	29.88	0.040
*k*_1_	/	0.1498	0.0094	0.1450	0.0126	0.0653	0.0016
*k*_2_	/	−0.948	0.2082	−0.951	** 0.2845	0.0000	*FIXED*
*p*_2_	/	−0.0080	0.00469	−0.0098	0.00005	−0.0099	0.00000
*p*_1_	/	0.002259	0.00027	0.00197	0.00015	0.0000	*FIXED*
RX_S-Z_	°	3.979	** 0.816	4.171	0.005	4.169	0.0043
RY_S-Z_	°	−14.883	0.022	−14.878	0.001	−14.879	0.0026
RZ_S-Z_	°	−134.946	** 0.307	−135.04	0.003	−135.043	0.0039
X_S-Z_	mm	112.83	** 1.300	112.66	0.013	112.64	0.017
Y_S-Z_	mm	5.35	** 1.317	5.83	0.014	5.81	0.025
Z_S-Z_	mm	219.80	** 1.010	220.54	0.079	220.50	0.082
DEV	mm	0.1081	0.00022	0.1081	0.00010	0.1090	0.00020

Parameter where *FIXED* is written instead of standard deviation was not optimized.

**Table 2:** Average values of optimized transformation parameters and respective standard deviations for N = 5 repeated calibrations where the hand–eye configuration was randomly varied.

Parameter	Unit	Average	Standard Deviation
X_B-R_	mm	106.89	0.026
Y_B-R_	mm	1152.88	0.043
Z_B-R_	mm	−239.70	0.118
RX_B-R_	°	−1.126	0.0031
RY_B-R_	°	0.462	0.0144
RZ_B-R_	°	0.517	0.0134
*f*	mm	8.387	0.0175
*c*_U_	pix	329.0	*FIXED*
*c*_V_	pix	247.0	*FIXED*
*α*	°	12.079	0.0078
*θ*	°	0.0	*FIXED*
*L*	mm	29.878	0.0384
*k*_1_	/	0.0656	0.0024
*k*_2_	/	0.0	*FIXED*
*p*_2_	/	0.0	*FIXED*
*p*_1_	/	−0.0100	0.0003

**Table 3:** Position-measurement uncertainty of the second object (N = 5).

Parameter	Average ^1^	Standard Deviation ^1^
X_PM_	1061.24	0.092
Y_PM_	44.49	0.038
Z_PM_	−159.66	0.094
Euclidean dist.	1074.10	0.107

^1^ All values are in mm.

**Table 4:** Contributions of each subsystem to the combined standard uncertainty for separate directions and Euclidean distance.

Subsystem	${u_{c{.X}_{R}}}^{1}$	${u_{c{.Y}_{R}}}^{1}$	${u_{c{.Z}_{R}}}^{1}$	${u_{c.\mathrm{Euclid}}}^{1}$
Profile detection	0.0000	0.0612	0.0586	0.0847
Robot positioning	0.1000	0.1000	0.1000	0.1732
3D transformation	0.0064	0.0054	0.0083	0.0118
Combined	0.1002	0.1174	0.1162	0.1932

^1^ All values are in mm.

**Table 5:** Comparison of the calibration results in the case of using a 5-axis CNC machine and robot arm.

Parameter	5-Axis CNC	Robot Arm
Average (DEV)	0.051	0.105
Standard deviation (X_PM_) ^1^	0.031	0.092
Standard deviation (Y_PM_) ^1^	0.013	0.038
Standard deviation (Z_-PM_) ^1^	0.032	0.094
Euclidean dist. ^1^	0.046	0.107

^1^ All values are in mm.

## Data Availability

The data presented in this study are available on request from the corresponding author.

## Appendix A

Table A1 shows an example of initial values of optimized parameters, corresponding weights which are used in normalization (see Equation (15)) and randomization intervals for parameters that are used during searching of the global minimum. These intervals are defined only for some parameters and represent a plus/minus value around the current optimal values (see Equation (16)).

**Table A1:** Initial values of optimized transformation parameters, their weights and randomization intervals.

Parameter	Initial Value	Weight	Randomization Interval	Unit
X_B-R_	107.30	1.0	0.0	mm
Y_B-R_	1153.00	1.0	0.0	mm
Z_B-R_	−239.30	1.0	0.0	mm
RX_B-R_	0.000	1.0	0.0	°
RY_B-R_	0.000	1.0	0.0	°
RZ_B-R_	0.000	1.0	0.0	°
*f*	8.00	0.1	0.1	mm
*c*_U_	329.5	0.1	0.0	pix
*c*_V_	247.0	0.1	0.0	pix
*α*	12.0	0.01	0.1	°
*θ*	0.0	0.01	0.0	°
*L*	30.0	0.01	0.1	mm
*k*_1_	0.0	0.01	0.0	/
*k*_2_	0.0	0.01	0.0	/
*p*_2_	0.0	0.001	0.0	/
*p*_1_	0.0	0.001	0.0	/
RX_S-Z_	7.600	0.1	1.0	°
RY_S-Z_	−12.000	0.1	1.0	°
RZ_S-Z_	130.000	0.1	1.0	°
X_S-Z_	115.00	1.0	1.0	mm
Y_S-Z_	0.00	1.0	1.0	mm
Z_S-Z_	220.00	1.0	1.0	mm

Table A2 presents sensitivity coefficients used in Equations (12), (14) and (15), which describe how the output measurands (X_R_, Y, and Z_R_) varies with the change of the transformation parameters. The coefficients were calculated according to GUM [33] recommendation using numerical simulation of small changes in transformation parameters.

**Table A2:** Initial values of optimized transformation parameters, their weights and randomization intervals.

Parameter (*C*_i_)	$\frac{\partial X_{R}}{\partial C_{i}}$	$\frac{\partial Y_{R}}{\partial C_{i}}$	$\frac{\partial Y_{R}}{\partial C_{i}}$	Unit
X_B-R_	−1.0 × 10^0^	−8.8 × 10^−3^	8.2 × 10^−3^	/
Y_B-R_	9.0 × 10^−3^	−1.0 × 10^0^	2.0 × 10^−2^	/
Z_B-R_	−8.0 × 10^−3^	−2.0 × 10^−2^	−1.0 × 10^0^	/
RX_B-R_	−2.9 × 10^−4^	6.6 × 10^−2^	3.1 × 10^−2^	°/mm
RY_B-R_	−6.6 × 10^−2^	−5.7 × 10^−4^	1.2 × 10^−2^	°/mm
RZ_B-R_	−3.1 × 10^−2^	−1.3 × 10^−2^	0.0 × 10^0^	°/mm
*f*	4.9 × 10^−2^	3.4 × 10^−1^	2.9 × 10^0^	/
*c*_U_	−1.1 × 10^−1^	7.9 × 10^−4^	2.0 × 10^−3^	pix/mm
*c*_V_	9.8 × 10^−3^	6.8 × 10^−2^	5.9 × 10^−1^	pix/mm
*α*	7.1 × 10^−2^	4.4 × 10^−1^	3.8 × 10^0^	°/mm
*θ*	−1.3 × 10^−2^	−8.2 × 10^−2^	−7.1 × 10^−1^	°/mm
*L*	−1.3 × 10^−2^	−4.4 × 10^−2^	−3.8 × 10^−1^	/
*k*_1_	−8.3 × 10^0^	−4.5 × 10^−1^	−1.7 × 10^−1^	1/mm
*k*_2_	−3.7 × 10^−1^	2.8 × 10^−3^	6.8 × 10^−3^	1/mm
*p*_2_	1.8 × 10^−3^	3.7 × 10^−1^	−4.4 × 10^−2^	1/mm
*p*_1_	−9.6 × 10^−2^	2.8 × 10^−2^	−1.3 × 10^−3^	1/mm
RX_S-Z_	7.1 × 10^−1^	−7.0 × 10^−1^	−6.1 × 10^−2^	°/mm
RY_S-Z_	−7.0 × 10^−1^	−7.0 × 10^−1^	−1.4 × 10^−1^	°/mm
RZ_S-Z_	5.6 × 10^−2^	1.4 × 10^−1^	−9.9 × 10^−1^	°/mm
X_S-Z_	−1.0 × 10^0^	−8.8 × 10^−3^	8.2 × 10^−3^	/
Y_S-Z_	9.0 × 10^−3^	−1.0 × 10^0^	2.0 × 10^−2^	/
Z_S-Z_	−8.0 × 10^−3^	−2.0 × 10^−2^	−1.0 × 10^0^	/
